# *In Silico* Identification of Protein Disulfide Isomerase Gene Families in the *De Novo* Assembled Transcriptomes of Four Different Species of the Genus *Conus*

**DOI:** 10.1371/journal.pone.0148390

**Published:** 2016-02-09

**Authors:** Andrea Figueroa-Montiel, Marco A. Ramos, Rosa E. Mares, Salvador Dueñas, Genaro Pimienta, Ernesto Ortiz, Lourival D. Possani, Alexei F. Licea-Navarro

**Affiliations:** 1 Departamento de Innovación Biomédica, Centro de Investigación y Estudios Superiores de Ensenada (CICESE), Ensenada, Baja California, México; 2 Facultad de Ciencias Químicas e Ingeniería, Universidad Autónoma de Baja California, Tijuana, Baja California, México; 3 Departamento de Medicina Molecular y Bioprocesos, Instituto de Biotecnología, Universidad Nacional Autónoma de México, Cuernavaca, Morelos, México; The University of Texas at El Paso, UNITED STATES

## Abstract

Small peptides isolated from the venom of the marine snails belonging to the genus *Conus* have been largely studied because of their therapeutic value. These peptides can be classified in two groups. The largest one is composed by peptides rich in disulfide bonds, and referred to as conotoxins. Despite the importance of conotoxins given their pharmacology value, little is known about the protein disulfide isomerase (PDI) enzymes that are required to catalyze their correct folding. To discover the PDIs that may participate in the folding and structural maturation of conotoxins, the transcriptomes of the venom duct of four different species of *Conus* from the peninsula of Baja California (Mexico) were assembled. Complementary DNA (cDNA) libraries were constructed for each species and sequenced using a Genome Analyzer Illumina platform. The raw RNA-seq data was converted into transcript sequences using Trinity, a *de novo* assembler that allows the grouping of reads into contigs without a reference genome. An N50 value of 605 was established as a reference for future assemblies of *Conus* transcriptomes using this software. Transdecoder was used to extract likely coding sequences from Trinity transcripts, and PDI-specific sequence motif “APWCGHCK” was used to capture potential PDIs. An *in silico* analysis was performed to characterize the group of PDI protein sequences encoded by the duct-transcriptome of each species. The computational approach entailed a structural homology characterization, based on the presence of functional Thioredoxin-like domains. Four different PDI families were characterized, which are constituted by a total of 41 different gene sequences. The sequences had an average of 65% identity with other PDIs. Using MODELLER 9.14, the homology-based three-dimensional structure prediction of a subset of the sequences reported, showed the expected thioredoxin fold which was confirmed by a “simulated annealing” method.

## Introduction

The genus *Conus* comprises over 700 species of carnivorous gastropods that lack the common radula present in the non-carnivorous gastropod species, instead have harpoon-like teeth for venom delivery. Also, the presence of a specialized apparatus for injection of venom enables them to quickly subdue fast-moving preys, such as other mollusks, worms, or even fish [1]. The venom of these organisms is mainly constituted by small and well-structured peptides, varying from 8 to 50 amino acid residues. During a long period of time it was believed that each species of *Conus* produced 50 to 200 different peptides, however improvements in mass spectrometry techniques increased this initial estimation about 10-fold [2]. The vast biodiversity of peptides in the venom of *Conus* snails make these organisms an excellent source of molecules with pharmacotherapeutic potential. This is particularly true in the neurosciences, where many of these peptides are being used for therapeutic purposes or as biochemical reagents [3]. Based on the content of disulfide bonds, peptides from *Conus* venom can be classified as conopeptides, if they are poor in disulfide bonds, or as conotoxins, if they are rich in disulfide bonds (where the Cys residues appear at high frequency, with many instances of multiple and adjacent pairs) [4]. Conotoxins represent the major content of the *Conus* venom and like many other eukaryotic polypeptides with a specific pattern of disulfide bonding require the assistance of a complex folding machinery to attain their functionally active three-dimensional conformation [5]. Although the folding pathway of conotoxins has not been well characterized, it has been proposed that disulfide oxidoreductase, such as the protein disulfide-isomerase (PDI), plays an important role in their oxidative folding [6].

Human PDI, recognized as a “typical” PDI, is one of the most extensively studied member of the PDI family and therefore it is used as baseline in the analysis of other PDIs. This 55 kDa protein exhibits a dual function: it catalyzes disulfide bond formation in polypeptide substrates, through its oxidase activity, and facilitates the rearrangement of incorrect disulfide bonds, through its isomerase activity [7]. A “typical” PDI consists of five linked and discrete domains (a, b, b', a' and c) [8]. The enzymatically active “a domains” are roughly 100 amino acid residues long and exhibit high homology to the prokaryotic protein thioredoxin (thus named thioredoxin-like domains). Furthermore, these domains share several structural and functional features, including the independent active CGHC motif (crucial for redox activity) [9,10] that has been reported that can be extended to an 8-residue sequence (APWCGHCK) [11,12]. Although they are enzymatically inactive, the “b domains” share high sequence similarity to each other but not to thioredoxin, despite the fact that they are capable of adopting a thioredoxin-like folding pattern [7]. Even though all “a and b domains” can contribute to the binding of misfolded proteins, the b’ domain provides the primary binding site [13]. Whilst the “c domain”, located near the C-terminal, is rich in acidic residues and has a high Ca^2+^-binding capacity [14]. As an ER-resident protein, PDI has a signal peptide at its N-terminus and the canonical sequence KDEL, ER-retention/retrieval tag, at its C-terminus region [15].

Over the last 50 years, the structural and functional characterization of peptide components of the venom from different species of *Conus* has favored both the gain of relevant scientific knowledge and the identification of novel molecules with therapeutic potential [1]. However, this has left an obscure gap in our knowledge about other components of the venom gland, including the chaperone machinery responsible for the folding and maturation of functional bioactive peptides. For instance, to date, there are just 6 nucleotide sequences encoding PDI homologs of *Conus* annotated in the NCBI Genbank database. Interestingly, Safavi-Hemami *et al*. (2011) identified, by a proteomics approach, a large number of PDI isoforms in the venom glands of *Conus novaehollandiae* and *Conus victoria*; demonstrating the co-localization of the chaperone machinery required for oxidative folding and the polypeptides that demand such process [16]. Hence, a thorough characterization of the molecular interaction between different PDI isoforms and distinct conotoxins families will provide further insights regarding the biodiversity of toxins in *Conus* snails [16].

Unlike the genome, which represents the whole gene content of an organism, the transcriptome depicts the complete set of actively transcribed genes at certain time and space. Therefore an RNA sequencing (RNA-seq) approach is particularly abundant in information about the mRNAs (and by inference the proteins) produced in a precise tissue. This is particularly important for venomous organisms [17], such as *Conus* snails, where production and storage of venom are restricted to a specific organ–the venom gland; which because of its shape, in the case of the *Conus* genus, is known as the venom duct. Furthermore, one hurdle in transcriptomic researches is when the species studied lacks a sequenced reference genome, typically needed for transcriptome assembly and gene model annotation (especially at the isoform level). This is the case of the *Conus* genus, where due to the lack of publicly available reference genomes, the bioinformatics pipeline for transcriptome assembly calls for specialized bioinformatics strategies, capable of *de novo* transcript assembly.

Here, we focused on four species of *Conus* (*C*. *californicus*, *C*. *mahogany*, *C*. *regularis*, and *C*. *ximenes*) that inhabit two distinctive areas of the peninsula of Baja California (Northwestern of Mexico). With the exception of *C*. *californicus*, the venom of the other species has been less explored, therefore, we assume that the information obtained here will be valuable for both comparative venom analyses and *Conus* toxinology, since most studies have been biased to species that inhabit Indo-Pacific areas [18–20]. High quality Illumina RNA-seq reads were used for *de novo* assembly of transcriptomes from venom ducts of the four *Conus* snails examined. The transcript information obtained was used for data-mining in order to identify putative PDI enzymes, focusing the efforts on the conserved amino acids of the motif “APWCGHCK” and using the “typical PDI” as a reference. Interestingly, we found a PDI family in each transcriptome assembly, indicating that the four species of *Conus* contain a set of proteins involved in oxidative folding. Furthermore, it has been well established that conotoxins are being expressed in the venom ducts of all *Conus*. These species are no different: several conotoxins were found in their transcriptomes and will be presented in a latter work. Therefore, the presumed co-localization of PDI enzymes and conotoxins suggests a dependence of the latter to a proper arrangement of disulfide bonds to adopt its active conformation.

## Materials and Methods

### Specimen collection and total RNA extraction

The specimens were collected in two separate locations of the Baja California Peninsula (Mexico), when the ocean tide was low. *C*. *californicus*, which is an atypical member of the genus, was collected in the cold waters of the Pacific side of the peninsula (40 km north of Ensenada). *C*. *mahogany*, *C*. *regularis*, and *C*. *ximenes* were collected in the southeast and warmer side of the peninsula (544 km south of Ensenada, Bahía de Los Angeles), all the species were identified by means of mitochondrial 16S sequence. The venom duct was dissected from each specimen under RNAse-free conditions (to assure the highest quality) and pooled in a single tube according to the species. RNA was isolated using the SV Total RNA Isolation System (Promega) following the protocol provided by the manufacturer. Briefly, the dissected venom ducts were manually macerated to homogeneity with a Kontes microtube pellet pestle rod (Daigger) in a 1.5 mL microtube with the provided RNA Lysis Buffer. After dilution with the RNA Dilution Buffer the sample was heated at 70°C for 3 minutes, then centrifuged to discard all cellular debris. The cleared lysate was mixed with 95% ethanol and transferred to one of the spin baskets supplied by the kit. After washing with the RNA Wash Solution, the sample was treated with the provided DNAse for 15 min and then washed twice with the RNA Wash Solution. After centrifugation, the total RNA was recovered in Nuclease-Free Water. Following verification of RNA quality and quantity by determining the absorbance ratio 260/280 (1.8–2.0), RNA integrity was reconfirmed using a 2100 Bioanalyzer instrument (Agilent Technologies).

### RNA-seq library and venom duct transcriptome assembly

A complementary DNA (cDNA) library was constructed from each *Conus* sample using the Illumina TruSeq Stranded mRNA Sample Preparation Kit, following the protocol provided by the supplier. Automated DNA sequencing was performed at the Core Facility of the Institute of Biotechnology (Cuernavaca, Mexico) with a Genome Analyzer IIx (Illumina), using a 72 bp paired-end sequencing scheme over cDNA fragments ranging in size of 200–400 bp. Each library consisted of two fastq files (forward and reverse reads), from which the adaptors were clipped-off. The quality of cleaned raw reads was assessed by means of the FastQC program (http://www.bioinformatics.bbsrc.ac.uk/projects/fastqc/). Since no reference genome is available for the examined *Conus* snails, short reads were assembled into contigs in a *de novo* fashion with Trinity software [21] (v. 2.0.3), using the standard protocol [22], executing the strand-specific parameter and normalizing reads. To weigh quality of the assembly, basic statistics for the number of genes and isoforms as well as the contiguity were obtained by running the TrinityStats.pl script. Bowtie2 [23], a tool for aligning sequencing reads to long reference sequences, was used to define the percentage of the raw reads that were mapped back to the assembled transcriptome. The Integrative Genomic Viewer (IGV) tool was used to visualize the assembled contigs [24].

### Bioinformatics analysis

All open reading frames (ORF) meeting the minimum length criteria for putative protein-coding sequences were extracted from the assembled transcripts using the Transdecoder utility, included in the Trinity software [21]. The structural motif “APWCGHCK” was next used as a probe to identify sequences encoding PDI-like polypeptides. The ORF sequences were named using the initials of the organisms followed by a consecutive number (*e*.*g*. first sequence found for *C*. *californicus* was designated “Cc01”). A survey of the protein database using the BLAST aligner at the NCBI server (http://BLAST.ncbi.nlm.nih.gov/Blast.cgi) revealed that all retrieved sequences were significantly similar to PDI counterparts, with an average of 65.4% (ranging from 44 to 98%), including several *Conus* PDI homologs. A PDI family was identified from the transcriptome assembly of each of the *Conus* snails examined. Using bioinformatics tools, each polypeptide was analyzed individually for structural features related to the function. The functional thioredoxin-like domains were predicted by homology using the Conserved Domain Database (CDD) search engine at the NCBI server (http://www.ncbi.nlm.nih.gov/Structure/cdd/wrpsb.cgi). The domain arrangement was obtained using the SMART (http://smart.embl-heidelberg.de) and Pfam (http://pfam.xfam.org) servers. Multiple sequence alignments were obtained initially using the Clustal Omega program at the EMBL-EBI server (http://www.ebi.ac.uk/Tools/msa/clustalo) and then edited with the software package Genious v. 4.8.5 [25]. A SignalP analysis was performed to identify the signal peptide cleavage site (http://www.cbs.dtu.dk/services/SignalP) and the molecular weight was calculated using EMBOSS Pepstats Platform (http://www.ebi.ac.uk/Tools/seqstats/emboss_pepstats).

### Prediction of *Conus* protein disulfide isomerase 3D structure

To identify a protein sequence with features of a “typical PDI” in each PDI family, predicted from the examined *Conus* snails, a pairwise alignment was performed for every member of each family using the human PDI as a template (PDB 4EKZ). The sequences with the highest similarity score (Cc03_i1, Cm05, Cr09 and Cx11) were selected to predict their three-dimensional (3D) structure by homology-based modelling. The 3D models were obtained using MODELLER v. 9.14, a program for comparative protein structure modelling [26], with the advanced procedure for multiple templates. After performing a BLAST of each selected sequence, 6 PDB files were chosen (4EKZ, 1MEK, 3UEM, 4JU5, 2KI8, 1X5C) based on the homology compared to the query sequences. The structural templates were obtained from the Protein Data Bank (http://www.rcsb.org/pdb/home/home.do).

The refining of the molecular modeled structures was conducted by molecular dynamic through the method of “simulated annealing” at 16 ns, standard pressure and constant temperature of 300°K, using the software NAMD Scalable Molecular Dynamics v. 2.10 of the Theorical and Computational Biophysics Group. This was performed in MIZTLI, a supercomputer owned by the National Autonomous University of Mexico (UNAM). The different conformation structures of the selected sequences were grouped based in their energetic stability and structural changes that undergo during the simulation with the purpose of selecting the best conformational structure with the longest existence time. Afterwards, an analysis of Ramachandran Plot was performed for the best conformational structure of each transcriptome, using the PROCHECK interactive server of the University of California, Los Angeles. These plots allowed us to compare the results of our models with a human PDI that has been well established by X-ray refraction [27].

## Results and Discussion

### Total RNA analysis

**Fig 1:**
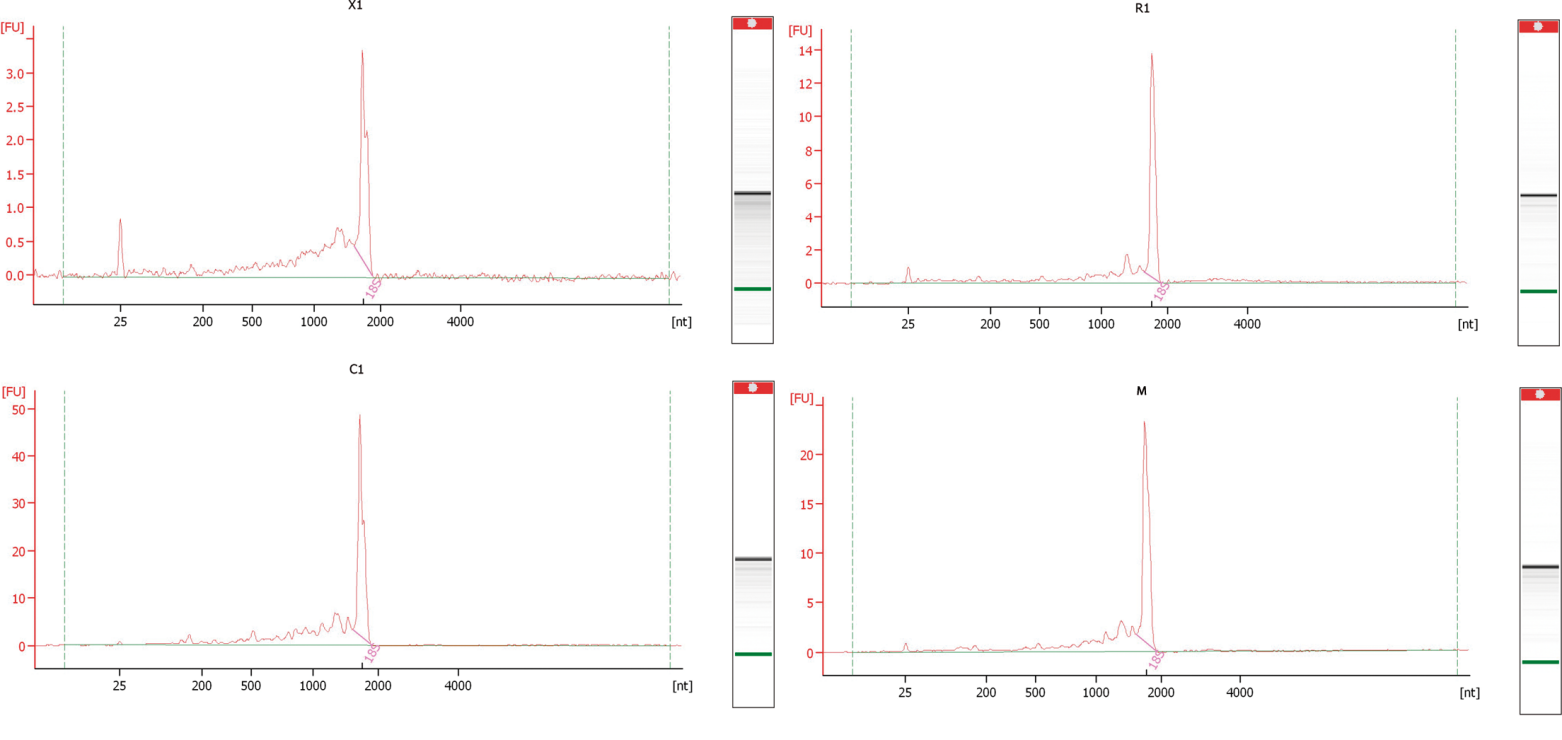
Electropherogram graphical results and gel-like image obtained with the Agilent Bioanalyzer for the RNA extractions of each venom duct. A single and clean ARN peak was obtained for *C*. *ximenes* (X1), *C*. *regularis* (R1), *C*. *californicus* (C1) and *C*. *mahogany* (M).

Extraction of total RNA from venom ducts of *C*. *californicus*, *C*. *mahogany*, *C*. *regularis*, and *C*. *ximenes* yielded 378, 44, 94, and 168 μg/mL, respectively. In all extractions, the graphical results obtained with the Agilent Bioanalyzer showed the absence of a peak corresponding to the 28S ribosomal RNA (Fig 1), making impossible to obtain the RNA Integrity Number (RIN). However, since a concentrated peak, sharp and without any signs of debris, was detected for the 18S ribosomal RNA, the integrity of the RNA sample was considered adequate for downstream applications. Interestingly, the lack of the 28S ribosomal RNA has been reported before in RNA extractions from insects, a phenomenon known as “the hidden break” [28]. In addition, this phenomenon has also been observed in RNA extractions from *C*. *episcopatus* [29].

### Sequencing and transcriptome assembly

Deep RNA-seq produced a vast number of reads from each *Conus* library (42’340,967 for *C*. *californicus*; 44’590,225 for *C*. *mahogany*; 50’360,289 for *C*. *regularis*; and 56’363,079 for *C*. *ximenes*). In addition, the FastQC report confirmed good quality reads. The complete set of reads for each library was independently used as input data during the assembly routine (with Trinity). The assembly performance was then evaluated by a set of basic statistics, including the N50 value. The summary of these statistics as well as the results of Bowtie2 alignments is shown in Table 1. The N50, a commonly used statistic value for assembly evaluation, is defined as the maximum length whereby at least 50% of the total assembled sequence resides in contigs of at least that length [21]. Unlike genome assemblies, maximizing N50 is not appropriate for transcriptomes; it is to be used as an index between closely related species. Also, it can be useful in confirming that the assembly succeeded. Our transcriptome assemblies showed a N50 value ranging from 490 to 746 (Table 1). A similar value (N50 = 478) was obtained by Lavergne *et al* (2015), using the same *de novo* assembler, for the transcriptome assembly of *C*. *episcopatus* [29], suggesting that the average transcript length, estimated by Trinity assembler, for the transcriptome of venom ducts from *Conus* snails is 605.6 ± 116.9. Moreover, the high percentage (more than 93%) of raw data aligned back to the contigs is a further evidence of the good quality of the assemblies, as judged by the Bowtie2 results (Table 1). So far, these are the highest values reported for *Conus* venom ducts transcriptomes, since the maximum values previously reported were 90.83% (using CLC Genomics Workbench assembler) and 87.35% (using Trinity assembler) [29].

**Table 1:** Summary of the basic statistics and Bowtie results.

	*C*. *californicus*	*C*. *mahogany*	*C*. *regularis*	*C*. *ximenes*
**Total transcripts**	24, 074	84,677	61,896	92, 433
**Average contig**	459.35	553.36	552.31	587.86
**N10**	1,477	2,439	2,254	2,411
**N50**	490	661	653	746
**%GC**	43.24	43.33	43.22	42.39
**%N**	0	0	0	0
**Bowtie2**	93.75%	95.63%	95.91%	95.38%

### *Conus* PDI-like family, structural analysis

As mention before, structurally, PDI proteins share the highly conserved motif “APWCGHCK”. So, using this as bait over the transcript database (with the Transdecoder program), a subset of PDI-like coding transcripts was extracted from each *Conus* venom duct transcriptome assembly. Table 2 shows the nomenclature assigned to each extracted transcript as well as the result of the comparative pairwise alignment obtained by BLAST.

**Table 2:** PDI-like coding transcripts extracted from each transcriptome assembly.

ORF nomenclature		Blast top hit	
Name	Completeness	ACCN (Protein, Organism)	ID (E)
Cc01	Partial	XP_011419975 (PDI A5-like, *Crassostrea gigas*)	54% (8e-70)
Cc02	Partial	ADZ76591 (PDI, *Conus eburneus*)	90% (0.0)
Cc03_i1	Complete	ADZ76591 (PDI, *Conus eburneus*)	68% (0.0)
Cc03_i4	Complete	ADZ76591 (PDI, *Conus eburneus*)	68% (0.0)
Cc04	Complete	ADZ76591 (PDI, *Conus eburneus*)	65% (0.0)
Cc05	Complete	XP_005100750 (PDI A3-like, *Aplysia californica*)	63% (0.0)
Cc06	Partial	ADZ76590 (PDI, *Conus virgo*)	80% (1e-50)
Cm01	Partial	EKC22564 (PDI A4, *Crassostrea gigas*)	64% (6e-60)
Cm02	Complete	AEE36485 (PDI 1, *Fenneropenaeus chinensis*)	51% (9e-134)
Cm03	Partial	ADZ76591 (PDI, *Conus eburneus*)	68% (0.0)
Cm04_i1	Complete	XP_011453191 (PDI A3-like, *Crassostrea gigas*)	65% (0.0)
Cm04_i2	Partial	XP_011453191 (PDI A3-like, *Crassostrea gigas*)	62% (2e-64)
Cm05	Complete	ADZ76593 (PDI, *Conus betulinus*)	86% (0.0)
Cm06	Partial	XP_002739058 (PDI A5-like, *Saccoglossus kowalevskii*)	51% (9e-33)
Cm07	Complete	XP_005108921 (PDI A6-like, *Aplysia californica*)	71% (0.0)
Cm08	Partial	XP_002732815 (PDI A4-like, *Saccoglossus kowalevskii*)	44% (3e-14)
Cm09	Partial	ABF48564 (PDI, *Conus marmoreus*)	71% (7e-42)
Cr01	Partial	GAA48005 (PDI A1, partial, *Clonorchis sinensis*)	60% (4e-85)
Cr02	Complete	XP_001602967 (probable PDI A6, *Nasonia vitripennis*)	64% (0.0)
Cr03	Partial	XP_002732815 (PDI A4-like, *Saccoglossus kowalevskii*)	50% (7e-35)
Cr04	Partial	EKC22564 (PDI A4, *Crassostrea gigas*)	71% (3e-53)
Cr05	Partial	AEE36485 (PDI 1, *Fenneropenaeus chinensis*)	57% (9e-41)
Cr06	Partial	XP_011429706 (P5-like, *Crassostrea gigas*)	48% (5e-80)
Cr07_i1	Complete	ADZ76591 (PDI, *Conus eburneus*)	65% (0.0)
Cr07_i2	Complete	ADZ76590 (PDI, *Conus virgo*)	67% (0.0)
Cr07_i3	Partial	ADZ76590 (PDI, *Conus virgo*)	66% (0.0)
Cr08	Complete	XP_005100750 (PDI A3-like, *Aplysia californica*)	64% (0.0)
Cr09	Complete	ADZ76591 (PDI, *Conus eburneus*)	95% (0.0)
Cr10	Partial	ADZ76593 (PDI, *Conus betulinus*)	98% (4e-76)
Cr11	Partial	XP_011679321 (PDI A5, *Strongylocentrotus purpuratus*)	51% (7e-30)
Cx01	Partial	GAA48005 (PDI A1, partial, *Clonorchis sinensis*)	62% (2e-111)
Cx02	Complete	ADZ76591 (PDI, *Conus eburneus*)	68% (0.0)
Cx03	Complete	XP_011453191 (PDI A3-like, *Crassostrea gigas*)	62% (0.0)
Cx04	Partial	XP_002739058 (PDI A5-like, *Saccoglossus kowalevskii*)	46% (1e-51)
Cx05	Partial	XP_008200981 (P5, *Tribolium castaneum*)	50% (4e-82)
Cx06	Partial	GAA48005 (PDI A1, partial, *Clonorchis sinensis*)	52% (5e-77)
Cx07	Partial	XP_005108921 (PDI A6-like, *Aplysia californica*)	73% (5e-136)
Cx08	Partial	ADZ76591 (PDI, *Conus eburneus*)	75% (1e-41)
Cx09	Partial	XP_005099456 (PDI A4-like, *Aplysia californica*)	58% (0.0)
Cx10	Partial	ABJ89816 (PDI ER-60, partial, *Clonorchis sinensis*)	58% (2e-113)
Cx11	Complete	ADZ76593 (PDI, *Conus betulinus*)	86% (0.0)

ACCN, Accession number; ID, identity; E, e-value; Cc, *C*. *californicus*; Cm, *C*. *mahogany*; Cr, *C*. *regularis*; Cx, *C*. *ximenes*.

Since PDI proteins share similar structural features, *e*.*g*., thioredoxin-like domains, the *Conus* counterparts were analyzed by comparative approaches using bioinformatics tools to get insights into their functional performance. Typically, the structure of PDI proteins comprises one to three functionally-active thioredoxin-like domains [30]. Interestingly, the highly conserved domain arrangement observed in “typical” PDI proteins (a-b-b´-a´) was found in some *Conus* PDI-like sequences, indicating that a crucial oxidoreductive enzyme involved in protein folding is present in venom ducts of these marine snails. The structural features of each *Conus* PDI-like family are described below.

#### *C*. *ximenes* PDI-like family

Eleven sequences were found in the *C*. *ximenes* venom duct transcriptome. Three of them with complete ORF (Cx02, Cx03, and Cx11) and the remaining with partial CDS (Table 2). The BLAST search revealed high identity degree with annotated PDI homologues, including some from species *Conus*. Moreover, the highest percentage of identity was observed between Cx11 and a PDI from *C*. *betulinus* (86%). The polypeptides encoded by the three complete ORF exhibit structural features that are prevalent among “typical” PDI (Table 3): a molecular weight within 55 and 60 kDa, the classical domain architecture (a-b-b´-a´), the conserved active site motif (CXXC) within each enzymatically active “a domain”, and a C-terminal tetrapeptide resembling the canonical ER-retention signal (KDEL). Although eight sequences represent partial CDS, the structural analysis of their incompletely-coded polypeptides showed some features that are worth to highlight (Table 3). All of them have at least one “a-like domain”, with their respective active site motif, and those with a full C-terminus (Cx01, Cx04, and Cx08-Cx10) exhibit a tetrapeptide resembling the ER-retention signal.

**Table 3:** Structural analysis features of the *C*. *ximenes* PDI-like family.

ORF	MW (kDa)	Domain Organization	Active Site Motif	ER-Retention Signal
Cx01	ND	(*)-b-a	CGHC	RDEL
Cx02	56.21	a-b-b´-a´	CGHC, CGHC	RDEL
Cx03	57.94	a-b-b´-a´	CGHC, CGHC	KSEL
Cx04	ND	(*)-a	CGHC	KDEL
Cx05	ND	(*)-a-b-b´-a´-(*)	CGHC, CGHC	ND
Cx06	ND	(*)-a-a´-(*)	CGHC, CGHC	ND
Cx07	ND	(*)-a-b-(*)	CGHC	ND
Cx08	ND	(*)-a	CGHC	KDEL
Cx09	ND	(*)-a	CGHC	RDEL
Cx10	ND	(*)-b-a	CGHC	KVDL
Cx11	56.06	a-b-b´-a´	CGHC, CGHC	RDEL

ND, not determined

(*), partial end-terminal sequence.

#### *C*. *regularis* PDI-like family

Thirteen PDI sequences were retrieved from *Conus regularis* venom duct transcriptome. The sequences that presented complete ORF were: Cr02, Cr07_i1, Cr07_i2, Cr08, and Cr09; while the remaining presented partial CDS (Table 2). After a BLAST search, a high degree of identity with annotated PDI homologues was revealed. Worth to note, we found three isoforms of Cr07 with high identity degree (65–67%) to PDI counterparts from *C*. *eburneus* and *C*. *virgo*. Also, the highest identity was observed between Cr09 and a PDI from *C*. *eburneus* (95%). The polypeptides Cr07_i1, Cr07_i2, Cr08, and Cr09 showed structural features characteristic of a “typical” PDI, which were described in the previous section (Table 4). Whilst the polypeptide encoded by the complete ORF named Cr02 shows the structural features of the PDI family member reported as P5/ERP5 (Table 4): a molecular weight around 48 kDa, two “a domains" (both containing the conserved the CXXC motif) and one “b domain” arranged in a particular organization (a-a´-b), and a stretch rich in acidic residues before the C-terminal ER-retention signal (KDEL). For the rest of the sequences (constituting partial CDS), the structural analysis showed some features that are worth describing (Table 4): all of them have at least one “a-like domain”, with their respective CXXC motif, and those with a full C-terminus (Cr06 and Cr10) exhibit a tetrapeptide resembling the ER-retention signal.

**Table 4:** Structural analysis features of the *C*. *regularis* PDI-like family,

ORF	MW (kDa)	Domain Organization	Active Site Motif	ER-Retention Signal
Cr01	ND	(*)-b-a-(*)	CGHC	ND
Cr02	47.47	a-a´-b	CGHC, CGHC	KDEL
Cr03	ND	(*)-a-(*)	CGHC	ND
Cr04	ND	(*)-a-(*)	CGHC	ND
Cr05	ND	(*)-a-(*)	CGHC	ND
Cr06	ND	(*)-a-a´	CGHC, CGHC	HTEL
Cr07_i1	55.35	a-b-b´-a´	CGHC, CKYC	KDEL
Cr07_i2	55.50	a-b-b´-a´	CGHC, CGHC	KDEL
Cr07_i3	ND	a-b-b´-a´-(*)	CGHC, CGHC	ND
Cr08	57.48	a-b-b´-a´	CGHC, CGHC	KTEL
Cr09	56.50	a-b-b´-a´	CGHC, CGHC	KDEL
Cr10	ND	(*)-a	CGHC	KDEL
Cr11	ND	(*)-a-(*)	CGHC	ND

ND, not determined

(*), partial end-terminal sequence.

#### *C*. *mahogany* PDI-like family

*C*. *mahogany* venom duct transcriptome presented ten PDI sequences. Only Cm02, Cm04_i1, Cm05, and Cm07 showed complete ORF (Table 2). All sequences revealed high identity degree with annotated PDI homologues, being the highest score observed between Cm05 and a PDI from *C*. *betulinus* (86%). Since we found two alleles of Cm06 (differing in residue 37), just one of them was used for structural analysis purposes. Also, two isoforms of Cm04 sharing high similarity degree (up to 65%) with a PDI protein from *Crassostrea gigas* were identified. Cm04_i1 and Cm05 (encoded by complete ORF) exhibit structural features that are prevalent among “typical” PDI; whereas the polypeptide encoded by the third complete ORF (Cm07) exhibits the structural features of the PDI family member reported as P5/ERP5 (Table 5). In addition, the polypeptide encoded by the fourth complete ORF (Cm02) displays the structural features of the PDI family member reported as TXNDC5/ERP46 (Table 5): a molecular weight around 46 kDa, three “a domains” (all containing the conserved the CXXC motif), and a C-terminal tetrapeptide resembling the ER-retention signal (KDEL). Even though six sequences contain partial CDS, the structural analysis of their incompletely-coded polypeptide showed some features that are worth to point out (Table 5): all of them have at least one “a-like domain”, with their respective CXXC motif, and those with a full C-terminus (Cm01 and Cm06) show the KDEL tetrapeptide as ER-retention signal.

**Table 5:** Structural analysis features of the *C*. *mahogany* PDI-like family.

ORF	MW (kDa)	Domain Organization	Active Site Motif	ER-Retention Signal
Cm01	ND	(*)-a	CGHC	KDEL
Cm02	43.90	a°-a-a´	CGHC, CGHC, CGHC	HTEL
Cm03	ND	a-b-b´-a´-(*)	CGHC, CGHC	ND
Cm04_i1	57.86	a-b-b´-a´	CGHC, CGHC	KSEL
Cm04_i2	ND	a-(*)	CGHC	ND
Cm05	56.03	a-b-b´-a´	CGHC, CGHC	RDEL
Cm06	ND	(*)-a	CGHC	KDEL
Cm07	47.86	a-a´-b	CGHC, CGHC	KDEL
Cm08	ND	a-(*)	CGHC	ND
Cm09	ND	(*)-a-(*)	CGHC	ND

ND, not determined

(*), partial end-terminal sequence.

#### *C*. *californicus* PDI-like family

Seven sequences were identified from the *C*. *californicus* venom duct transcriptome. Four of them with complete ORF (Cc03_i1, Cc03_i4, Cc04, and Cc05) and the remaining with partial CDS (Table 2). As occurred in the other transcriptomes, after BLAST search, all sequences revealed high identity degree with annotated PDI homologues (including some from species of *Conus*). Since four variants of Cc03 were found: three alleles (Cc03_i1, _i2, and _i3, differing in residues 288 and 387) and one isoform (Cc03_i4), just Cc03_i1 and Cc03_i4 were used for structural analysis purposes. Interestingly, the highest similarity value was observed between Cc03 variants and PDI from *C*. *eburneus* (68%). The polypeptides encoded by three complete ORF (Cc03_i1, Cc03_i4, and Cc05) show structural features that are prevalent among “typical” PDI (Table 6). The polypeptide encoded by the complete ORF denominated Cc04 displays the structural features of the PDI family member reported as P5/ERP5 (Table 6). The structural analysis of the three sequences containing partial CDS incompletely-coded polypeptide showed the same features as the incomplete sequences from the rest of the transcriptomes (Table 6); being Cc01 and Cc02 the ones that show a tetrapeptide with high resemblance to the canonical ER-retention signal.

**Table 6:** Structural analysis features of the *C*. *californicus* PDI-like family.

ORF	MW (kDa)	Domain Organization	Active Site Motif	C- terminal tetrapeptide
Cc01	ND	(*)-a-a´	CGHC, CGHC	KEEL
Cc02	ND	(*)-a-b-b´-a	CGHC	RDEL
Cc03_i1	56.27	a-b-b´-a´	CGHC, CGHC	KDEL
Cc03_i4	57.45	a-b-b´-a´	CGHC, CGHC	KDEL
Cc04	48.22	a-a´-b	CGHC, CGHC	KEEL
Cc05	55.73	a-b-b´-a	CGHC, CGHC	KTEL
Cc06	ND	(*)-a-(*)	CGHC	ND

ND, not determined

(*), partial end-terminal sequence.

### Biological significance of a PDI-like family in *Conus* snails

Although *C*. *californicus* is considered a highly divergent marine snail [31], our results show a high degree of similarity among the analyzed species. Moreover, the diversity of each PDI-like family reveals a glimpse of the intricate molecular chaperone machinery likely to be established in *Conus* snails. This complexity has been observed in higher organisms; for instance, 21 members have been reported for the human PDI family, varying in molecular weight, domain composition, tissue expression, and cellular processing [32].

On the other hand, conotoxins are not conventional peptides, due to their structured polypeptide backbone and the characteristic arrangement of cysteine residues (which are assembled into a particular disulfide bonding configuration, known as the “disulfide framework”) [33]. As previously known, the folding of proteins having cysteine residues requires an additional biochemical modification: the disulfide bond formation (oxidation), and the hydrolysis (reduction) or rearrangement (isomerization) of those incorrectly formed [30]. Conotoxins are no exception, even more, their high content of cysteine residues (up to 50% of all residues can be cysteines involved in disulfide bonding [33]) imposes a further challenge. Also, assuming that the variety of conotoxins in a venom duct can be hundreds or thousands, it is not surprising that the enzymes responsible for their correct folding show a wide complexity. Additionally, it is justified to suppose that only a subset of the *Conus* PDI-like family are able to catalyze efficient oxidative folding, as observed in other PDI families, such as the human family [30]. Moreover, our results provide further evidence regarding the diversity of PDI-like proteins found in venom ducts of species of *Conus* snails, supporting the results of previous studies [5,33]. Furthermore, since the native conformation of conotoxins depends on their correct “disulfide framework” (25 frameworks have been established so far [3]), a wide variety of PDI-like isoforms it’s likely needed in order to maintain the demand for multiple different conotoxins present in the venom duct.

### Alignments of PDI

**Fig 2:**
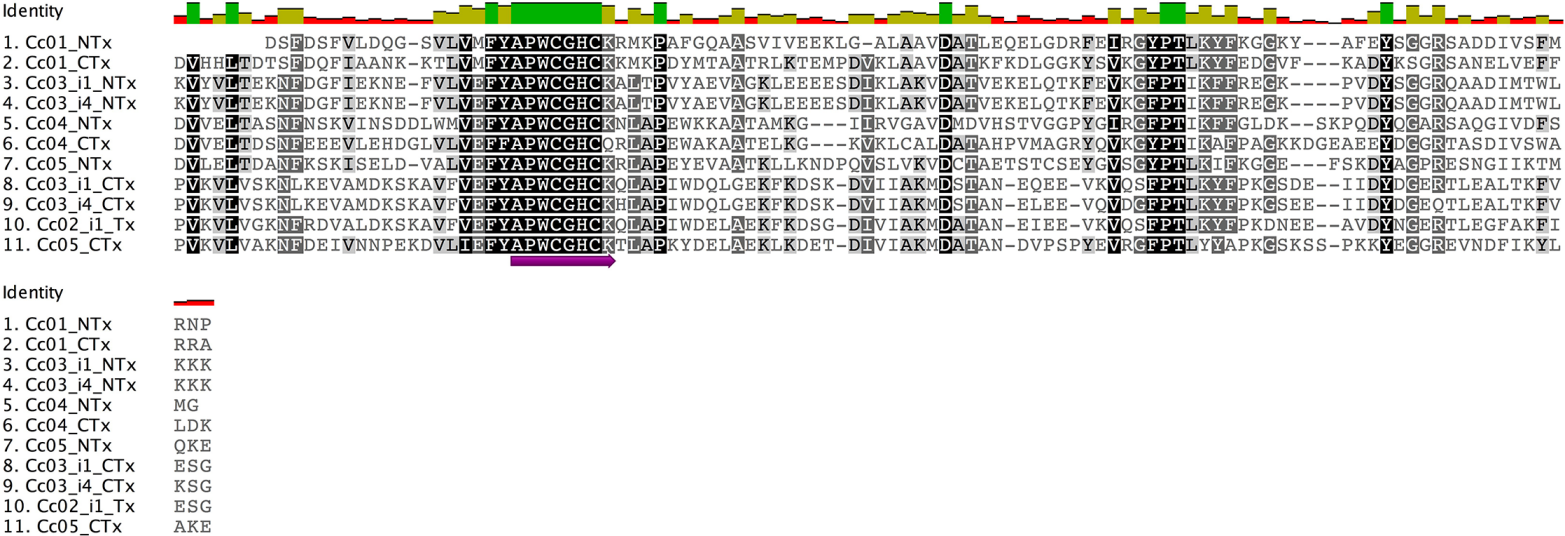
Sequence comparison of the *C*. *californicus* catalytic domains. Alignment of the a-like domains of *C*. *californicus* were made. It is shown that the APWCGHCK motif is clearly conserved, and in all cases it can be extended to FYAPWCGHCK. Cc04 presented two active domains: the CTx domain presented a Q instead of a K in the conserved motif. Another set of residues is clearly conserved at the end of the motif (GYPT) but is not big enough to use it as bait.

Despite of the fact that the structural motif “CGHC” is required in the enzymatically active domains (a and a’) for efficient oxidoreductase activity, an extended version of this motif (APWCGHCK), but less mention, has been established as conserved between PDI enzymes [11,12]. We confirmed a longer motif that can be successfully used as a probe to obtain *Conus* PDI-like sequences: FYAPWCGHCK which Knodler *et al* (1999) identified as F(Y/F)APWCGHCK [12]. Since a sequence of 7 residues has been established as the minimum probe length needed to provide unambiguous protein identification [34], our 10-residue sequence fulfills this requirement and, in some cases, it could be even be extended to a general 12-residue sequence (VEFYAPWCGHCK). It is worth mention that slightly variations of this extended active motif are found between species, where the bold letter is the amino acid that appears in the majority of the sequences: for *C*. *californicus* (Fig 2) it can be established as (**V**/I)(**E**/M)FYAPWCGHC(**K/**Q); for *C*. *ximenes* (S1 Fig) it is presented as (**V**/I)(**E**/M/K)(**F**/L)(F/**Y**/H)APWCGHCK; for *C*. *regularis* (S2 Fig) the motif is (V/I)(E/M/K)(F/L)(F/Y)APWCGHC(K/Q) and finally for C. mahogany (S3 Fig) it is shown as (**V**/I)(**E**/M/K)(**F**/L)(F/**Y**)APWCGHCK(**K**/Q).

### *Conus* PDI-like 3D structure prediction

**Fig 3:**
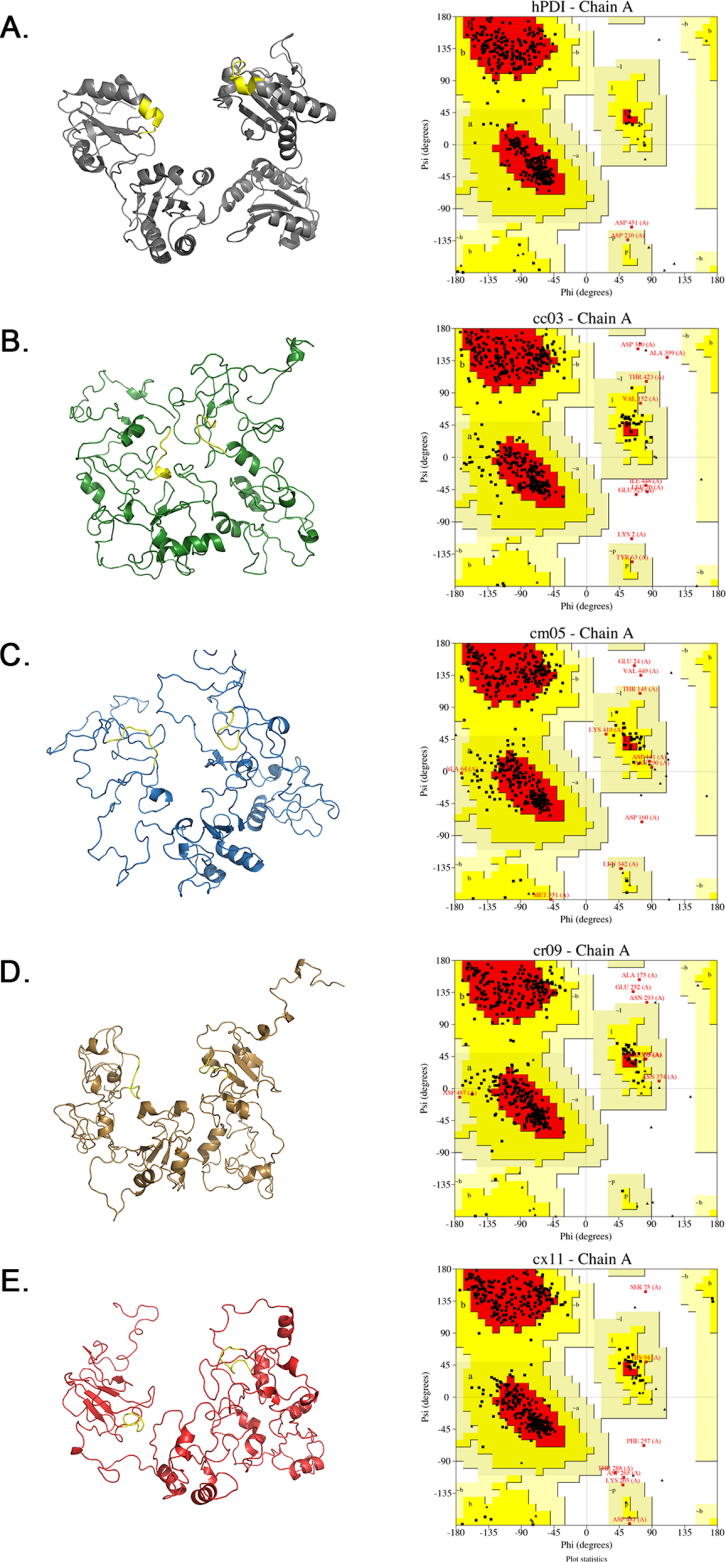
*Conus* PDI maintain the same protein folding as the human PDI documented in www.PDB.org. A) Crystal Structure and Ramachandran plot of reduced hPDI (4EKZ) show in grey. Refined model structure and their respectively Ramachandran plot of B) *C*. *californicus* (Cc03_i1) show in green, C) *C*. *mahogany* (Cm05) show in blue, D) *C*. *regularis* (Cr09) show in brown and E) *C*. *ximenes* (Cx11) show in red. The active motifs (APWCGCHCK) are indicated in yellow in all molecules.

From each transcriptome, the sequence with the highest similarity degree to the crystal-solved human PDI enzyme (PDB 4EKZ) was used as representative of each PDI-like family to predict a 3D model, and get insights into the structure-function relationship: Cc03_i1 for *C*. *californicus*, Cm05 for *C*. *mahogany*, Cr09 for *C*. *regularis*, and Cx11 for *C*. *ximenes*. The different conformation structures, after refining the molecular model, of the selected sequences are presented in Supporting Information Tables: Cc03_i1 (S1 Table), Cm05 (S2 Table), Cr09 (S3 Table) and Cx11 (S4 Table). As depicted in Fig 3, the inferred three-dimensional conformation displayed by PDI-like proteins from *Conus* snails suggest that they have a functional enzyme involved in oxidative folding of nascent polypeptides, as the common folding pattern adopted by thioredoxin-like domains found in active PDI enzymes was observed in the *Conus* PDI-like counterparts. All the refined 3D models of *Conus* PDI showed to have similarity to the structure obtain by experimental methods.The residue percentage of the most favoured region (A,B,L) for Cc03_i1 (80.2%), Cm05 (76.9%), Cr09 (81.2%) and Cx11(82.9%) are satisfactory, moreover are similar to the percentage of the human PDI obtain by X-ray diffraction (93.2%).

## Conclusions

A total of 41 different transcript sequences encoding PDI-like proteins were retrieved from the venom duct transcriptomes of four different species of *Conus* snails. To our knowledge, this is the closest approach intended to identify a PDI-like protein family from several species of *Conus*. Our structural results suggest that these marine snails have a PDI-like protein family that is necessary for the correct oxidative folding of the vast range of polypeptides that constitute their venom, including conotoxins. Furthermore, in order to extend our knowledge and get the full PDI-like family of each *Conus* snail studied, we propose the use of the sequence “FYAPWCGHCK” as a probe to get stringent conditions in further surveys. Finally, since the study of *Conus* venom has been focused on the discovery of new active molecules that could be use as therapeutics, we believe that in order to achieve a fruitful production of conotoxins, their study must go tête-à-tête with the understanding of their oxidative folding requirements.

## Supporting Information

S1 Fig Catalytic domains alignment of *C*. *ximenes*. (PDF)

S2 Fig Sequence comparison of the *C*. *regularis* catalytic domains. (PDF)

S3 Fig a-like sequences comparison of the PDI-family of *C*. *mahogany*. (PDF)

S1 Table PDI conformations of *C*. *californicus* (Cc03_i1). (PDF)

S2 Table PDI conformations of *C*. *mahogany* (Cm05). (PDF)

S3 Table PDI conformations of *C*. *regularis* (Cr10). (PDF)

S4 Table PDI conformations of *C*. *ximenes* (Cx12). (PDF)
