# Supplementary material for: In Silico Identification of Protein Disulfide Isomerase Gene Families in the De Novo Assembled Transcriptomes of Four Different Species of the Genus Conus
Source: PLoS One. 2016 Feb 9;11(2):e0148390. doi: 10.1371/journal.pone.0148390 (PMC4747531; doi:10.1371/journal.pone.0148390)
Supplement: S1 Fig — (PDF) [file pone.0148390.s001.pdf]

```
Identity

1. Cx05_NTx     ALVELTDDTFEDFIQT--GFHFVKFYAPWCGHCKRLAPTWEELAKSFA-DN-KQVSVSKVDCTVSTKLCTNQGVRGYPTLLLFNNGVKL---EQYQGSRSHEDLK
2. Cx05_CTx     AVIDLTEDSYEEGISG--GLTFVKFFAPWCGHCKRLAPTWEKLGTANK-D--KDITIARVDCTQHKSICDKESVS----L
3. Cx07_Tx      DVVEVTDSNFEKEVLEYDGLVMVEFYAPWCGHCKNLAPHWAKAATELK-G---KVKLCALDATVHTVMSNRYGVRGFPTIKVFPAGKKDGEAEEYDGGRTSSDIV
4. Cx02_NTx     KVYVLTEKNFDGFIQDN-EFVLVEFYAPWCGHCKALAPTYAEVAGKFE-DEDSNIKLAKVDATVEKALQAKFEVKGFPTIKFFRNGDP----IEYTGGRQASDII
5. Cx11_NTx     GVYVLTEKNFDAFVKEN-EFVLVEFYAPWCGHCKALAPEYSKAAETLE-GEKSNIKLGKVDATVESDLASKHGIRGYPTIKFFRDGTP----SDYSAGRQAEDIV
6. Cx06_Tx      GVAILTKDIFHSVIEEY-PYVMVEFYAPWCGHCKALKPEYEKVAGLLK-AELKDVLIAMVDATVESDLATEFGVMGYPTVKFRKNGVW----VDYQGERTSSAIV
7. Cx09_Tx2     AVVVLTKENFTDTVERE-ELMLVEFYAPWCGHCKSLAPKYEKAAKTLK-IGAQPIMLATVDATAETELASKYGVTGYPTLKVFRKGRD----FEYKGQRDQYGIV
8. Cx03_NTx     DVLEFTDSDFETKIKEH-KVALVEFYAPWCGHCKRLAPEYEVAATKLL-KNDPPVPLVKVDCTAETATCGKFGVSGYPTLKIFKDGEFS---KEYSGPREANGII
9. Cx04         DVHHLTDTSFDEFIAANK-RTLVMFYAPWCGHCKKMKPDYMTAATRLK--EVPDAKLAAVDVTKFKDLASKYSIKGYPTLKYFEDGAFK---ADYKSGRSAADLV
10. Cx02_CTx    PVKVLVSKNFKEVAMDKSKAVFVEFYAPWCGHCKQLAPVWDQLGEKFK--DSKDIIIAKMDSTANEM--EEVQIKSFPTLKYFPKGSDK--IVDYDGTRTLEALT
11. Cx08_Tx                            FYAPWCGHCKQLAPVWDQLGEKFK--DSKDIIIAKMDSTANEM--EEVQIKSFPTLKYFPKGSDK--IVDYDGTRTLEALT
12. Cx11_CTx    PVKVLVGKNFVDVALDKSKAVFVEFYAPWCGHCKQLAPIWDELGEKYK--DNSDIVIAKMDATANEI--EEVKVQSFPTLKYFPKDSDK--AVDYNGERTLEGFI
13. Cx01_Tx     PVRVLVGKNYNEVTQDESKAVFVKLYAPWCGHCKQLAPIWDELGEAFK--DRSDILIAKLDATANDV--DGLVVRAFPTLKYYPKGHQE--PVEYSGDRTLEAFK
14. Cx03_CTx    PVKVVVAKNFEDIVNNPEKDVLIEFYAPWCGHCKSLEPKYNELAEKLK--DETDIVIAKMDATANDPP-SQYEVRGFPTLYFAPKGSKSS-PKKYEGGREVNDFL
15. Cx10_Tx     AAVKVVGLTFDEIVNDPTKDVFIMFHAPWCGHCKQLMPKFESVAETLK--GEPSVVMAVYDATANEIF-PPYKVSGYPTLYFVPRNKKQA-PMPYQGAREAADLI

Identity

1. Cx05_NTx     GFITRK
2. Cx05_CTx
3. Cx07_Tx      QWAMEK
4. Cx02_NTx     NWLKKK
5. Cx11_NTx     NWLKKK
6. Cx06_Tx      SWIKRK
7. Cx09_Tx2     EYMNKQ
8. Cx03_NTx     KVMQKE
9. Cx04         DFFRTA
10. Cx02_CTx    KFVESG
11. Cx08_Tx     KFVESG
12. Cx11_CTx    KFLDSE
13. Cx01_Tx     KFVEND
14. Cx03_CTx    KYLAKE
15. Cx10_Tx     KFIAQE
```
