# Supplementary material for: In Silico Identification of Protein Disulfide Isomerase Gene Families in the De Novo Assembled Transcriptomes of Four Different Species of the Genus Conus
Source: PLoS One. 2016 Feb 9;11(2):e0148390. doi: 10.1371/journal.pone.0148390 (PMC4747531; doi:10.1371/journal.pone.0148390)
Supplement: S2 Fig — (PDF) [file pone.0148390.s002.pdf]

```
Identity

1. Cr02_NTx       GVVDLTPTNFHSKVINGDELWIVEFYAPWCGHCQSLVPEWKKAASALKG---IVKVGAVNADEHNALGGQYGVRGFPTIKFFGLDKFK--PQDYQGQRTAQGIVE
2. Cr02_CTx       DVVELTDSNFEKEVLGHDGLVMVEFYAPWCGHCKNLAPQWAKAASELKG---KVKLCALDATVHTVMANRYGVRGYPTIKTFPAGKKDGEAEDYDGGRTASDIVQ
3. Cr07_i1_NTx    NVYVLKQKNFD-NFIKENEFVLVEFYAPWCGHCKRLAPVYSEVAGKLVDEGSNIKLAKVDATVEKDLAGKFEVKGFPTMKFFINGEP----TDYTGGRQTSDFMV
4. Cr07_i3_NTx    NVYVLKQKNFD-NFIKENEFVLVEFYAPWCGHCKRLAPVYSEVAGKLVDEGSNIKLAKVDATVEKDLAGKFEVKGFPTMKFFINGEP----TDYTGGRQTSDFMV
5. Cr07_i2_NTx    NVYVLKQKNFD-NFIKENEFVLVEFYAPWCGHCQHLAPVYSEVAGKLVDEGSNIKLAKVDATVEKDLAGKFEVKGFPTMKFFINGEP----TDYTGGRQTSDFMV
6. Cr10_NTx       GVYVLTTKNFD-SFIAENEFVLVEFYAPWCGHCKALAPEYAKAATTLEEEKLNIKLGKVDATVEENLASKFEVRGYPTIKFFRKEKPDS-PSDYNGGRQAADIVN
7. Cr03_Tx        GVTVLSKGNFA-DFIESTETVLVEFYAPWCGHCKHLAPEYAAAAKELKQMDPPVLLAKVDATVESELAEEHEVTGYPTLKFFKKGKG
8. Cr05_Tx        DAVSYERDAFQ-SAIND-KKHFVMFFAPWCGHCKRLAPTWNELAKVYNNDESSVTIAKVDCTVETALCSEHDVTGYPTLKFFHKKADD--FQRYKGNRDLDSLKK
9. Cr06_CTx       AVMDLTADTFE-EAISE-GLTFVKFFAPWCGHCKRLAPTWEELARTSMG--KDIIIARVDCTQHKSVCDEQKVQGYPTLVLFQNGKRK--ND-YKGQRDLDSLQT
10. Cr06_NTx      DLTELTDDTFD-AFIQK-GFHFVKFYAPWCGHCKRLAPTWEELARSLAEN-KLVSVSKVDCTVSTKLCTSQGIRGYPTLILFNDG-EK--FEQYQGSRGLEDLKG
11. Cr09_NTx      DVLEFTDANFA-SEIKELDVALVEFYAPWCGHCKRLAPEYEVAATKLKKGDPPVSLVKVDCTAETKTCQEYGVSGYPTLKIFKGGEFA--KD-YQGPRESKGIIA
12. Cr11_Tx       LVPNFTDDNFD-VKIKERSFTLVMFYAPWCGHCKRFKPDFIAAGEQLIKETNNVGLALVDCTVHKKTCSDFGVNGYPTVKLFEDGKQT--HD-YDGSRD
13. Cr04_Tx       PVKVVVGKTFEKIVQDTKKDVLIELYAPWCGHCKKLEPVYKELAKKFKP-AKNLVIAKMDATANDVP-DEYKVEGFPTIYFAAADKKNV-PIKYEGGRALEDFEK
14. Cr08_CTx      PVKIAVAKNFEELVNDPEKDVLIEFYAPWCGHCKSLAPKYDELAEKLKD-ETDIVIAKMDATANDPP-SQYEVRGFPTIYFAPKGSKSS-PKKYEGGREVNDFIK
15. Cr07_i1_CTx   PVKVLVSKNFKEVAMDKSKAVFVEFYSPRCKYCKKFAPIWDQLGEKFMD-SKDIIIAKMDAKANEL--EEVQIKIYPTLKYFPKGSDE--IIDYDGKRTLEELTK
16. Cr07_i2_CTx   PVKVLVGKNFKEVAMDKSKAVFVEFYAPWCGHCKKLAPIWDQLGEKFKD-SKDIVIAKMDSTANEL--EEVEIRSFPTLKYFPKGSDE--IIEYDGGRTLEELTK
17. Cr09_CTx      PVKVLVGKNFKGVAMDKSKAVFVEFYAPWCGHCKQLAPIWDELGEKYKD-SKDIVVAKMDATANEI--EEVKVQSFPTLKYFPKDSEE--AVDYNGERTLDAFVK
18. Cr10_Tx       PVKVLVGKNFKGVAMDKSKAVFVEFYAPWCGHCKQLAPIWDELGEKYKD-SKDIVVAKMDATANEI--EEVKVQSFPTLKYFPKDSEE--AVDYNGERTLDAFVK

Identity

1. Cr02_NTx       FSMNQ
2. Cr02_CTx       WAMEK
3. Cr07_i1_NTx    WLKKK
4. Cr07_i3_NTx    WLKKK
5. Cr07_i2_NTx    WLKKK
6. Cr10_NTx       WLKKK
7. Cr03_Tx
8. Cr05_Tx        FVDEQ
9. Cr06_CTx       FVDSH
10. Cr06_NTx      FVTGK
11. Cr09_NTx      TMQKE
12. Cr11_Tx
13. Cr04_Tx       FLKEK
14. Cr08_CTx      YLAKE
15. Cr07_i1_CTx   FVESG
16. Cr07_i2_CTx   FVEG
17. Cr09_CTx      FLESG
18. Cr10_Tx       FLESG
```
