# Supplementary material for: In Silico Identification of Protein Disulfide Isomerase Gene Families in the De Novo Assembled Transcriptomes of Four Different Species of the Genus Conus
Source: PLoS One. 2016 Feb 9;11(2):e0148390. doi: 10.1371/journal.pone.0148390 (PMC4747531; doi:10.1371/journal.pone.0148390)
Supplement: S3 Fig — (PDF) [file pone.0148390.s003.pdf]

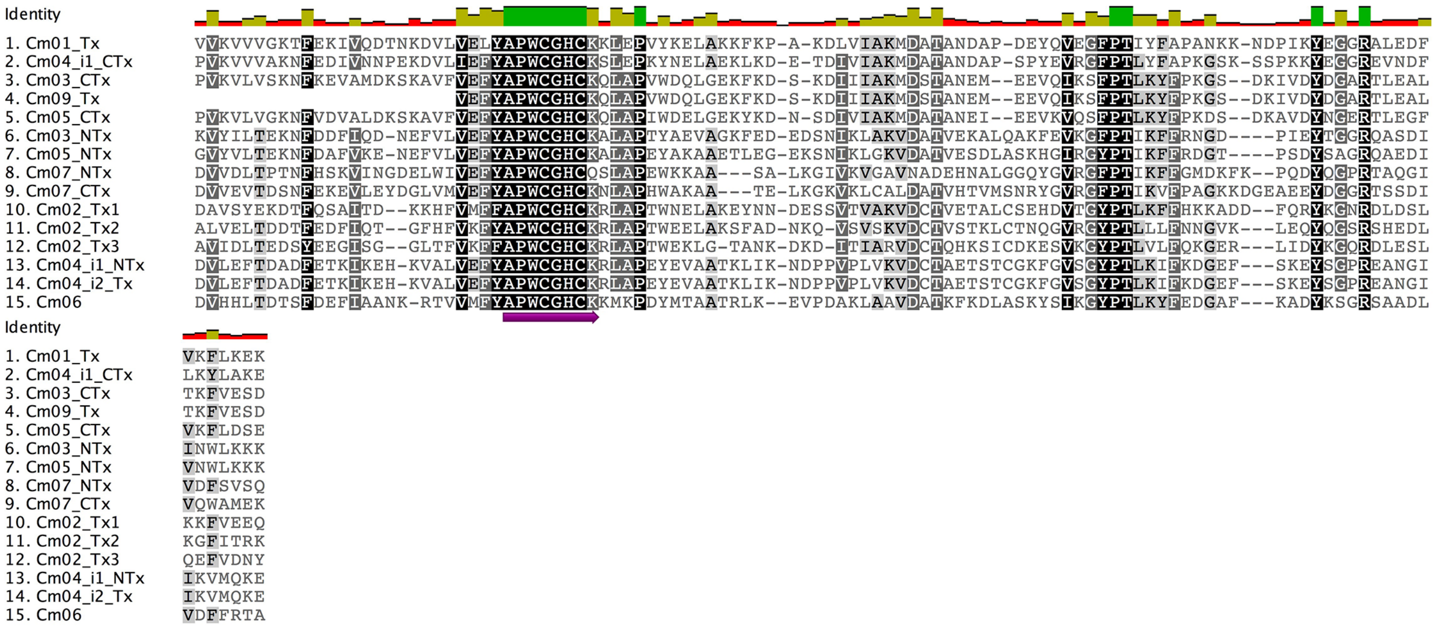
Identity
1. Cm01_Tx VVKVVVGKTFEKIVQDTNKDVLVELYAPWCGHCKKLEPVYKELAKKFKP-A-KDLVIAKMDATANDAP-DEYQVEGFPTIYFAPANKK-NDPIKYEGGRALEDF
2. Cm04_i1_CTx PVKVVVAKNFEDIVNNPEKDVLIEFYAPWCGHCKSLEPKYNELAEKLKD-E-TDIVIAKMDATANDAP-SPYEVRGFPTLYFAPKGSK-SSPKKYEGGREVNDF
3. Cm03_CTx PVKVLVSKNFKEVAMDKSKAVFVEFYAPWCGHCKQLAPVWDQLGEKFKD-S-KDIIIAKMDSTANEM--EEVQIKSFPTLKYFPKGS--DKIVDYDGARTLEAL
4. Cm09_Tx VEFYAPWCGHCKQLAPVWDQLGEKFKD-S-KDIIIAKMDSTANEM--EEVQIKSFPTLKYFPKGS--DKIVDYDGARTLEAL
5. Cm05_CTx PVKVLVGKNFVDVALDKSKAVFVEFYAPWCGHCKQLAPIWDELGEKYKD-N-SDIVIAKMDATANEI--EEVKVQSFPTLKYFPKDS--DKAVDYNGERTLEGF
6. Cm03_NTx KVYILTEKNFDDFIQD-NEFVLVEFYAPWCGHCKALAPTYAEVAGKFED-EDSNIKLAKVDATVEKALQAKFEVKGFPTIKFFRNGD----PIEYTGGRQASDI
7. Cm05_NTx GVYVLTEKNFDAFVKE-NEFVLVEFYAPWCGHCKALAPEYAKAAETLEG-EKSNIKLGKVDATVESDLASKHGIRGYPTIKFFRDGT----PSDYSAGRQAEDI
8. Cm07_NTx DVVDLTPTNFHSKVINGDELWIVEFYAPWCGHCQSLAPEWKKAA---SA-LKGIVKVGAVNADEHNALGGQYGVRGFPTIKFFGMDKFK--PQDYQGPRTAQGI
9. Cm07_CTx DVVEVTDSNFEKEVLEYDGLVMVEFYAPWCGHCKNLAPHWAKAA---TE-LKGKVKLCALDATVHTVMSNRYGVRGFPTIKVFPAGKKDGEAEEYDGGRTSSDI
10. Cm02_Tx1 DAVSYEKDTFQSAITD--KKHFVMFFAPWCGHCKRLAPTWNELAKEYNN-DESSVTVAKVDCTVETALCSEHDVTGYPTLKFFHKKADD--FQRYKGNRDLDSL
11. Cm02_Tx2 ALVELTDDTFEDFIQT--GFHFVKFYAPWCGHCKRLAPTWEELAKSFAD-NKQ-VSVSKVDCTVSTKLCTNQGVRGYPTLLLFNNGVK---LEQYQGSRSHEDL
12. Cm02_Tx3 AVIDLTEDSYEEGISG--GLTFVKFFAPWCGHCKRLAPTWEKLG-TANK-DKD-ITIARVDCTQHKSICDKESVKGYPTLVLFQKGER---LIDYKGQRDLESL
13. Cm04_i1_NTx DVLEFTDADFETKIKEH-KVALVEFYAPWCGHCKRLAPEYEVAATKLIK-NDPPVPLVKVDCTAETSTCGKFGVSGYPTLKIFKDGEF---SKEYSGPREANGI
14. Cm04_i2_Tx DVLEFTDADFETKIKEH-KVALVEFYAPWCGHCKRLAPEYEVAATKLIK-NDPPVPLVKVDCTAETSTCGKFGVSGYPTLKIFKDGEF---SKEYSGPREANGI
15. Cm06 DVHHLTDTSFDEFIAANK-RTVVMFYAPWCGHCKKMKPDYMTAATRLK--EVPDAKLAAVDATKFKDLASKYSIKGYPTLKYFEDGAF---KADYKSGRSAADL
Identity
1. Cm01_Tx VKFLKEK
2. Cm04_i1_CTx LKYLAKE
3. Cm03_CTx TKFVESD
4. Cm09_Tx TKFVESD
5. Cm05_CTx VKFLDSE
6. Cm03_NTx INWLKKK
7. Cm05_NTx VNWLKKK
8. Cm07_NTx VDFSVSQ
9. Cm07_CTx VQWAMEK
10. Cm02_Tx1 KKFVEEQ
11. Cm02_Tx2 KGFITRK
12. Cm02_Tx3 QEFVDNY
13. Cm04_i1_NTx IKVMQKE
14. Cm04_i2_Tx IKVMQKE
15. Cm06 VDFFRTA
