# Supplementary material for: In Silico Identification of Protein Disulfide Isomerase Gene Families in the De Novo Assembled Transcriptomes of Four Different Species of the Genus Conus
Source: PLoS One. 2016 Feb 9;11(2):e0148390. doi: 10.1371/journal.pone.0148390 (PMC4747531; doi:10.1371/journal.pone.0148390)
Supplement: S1 Table — (PDF) [file pone.0148390.s004.pdf]

S1 Table. PDI conformations of *C. californicus* (Cc03_i1).

Conformation		Number of structures	Existence time (ns)
1	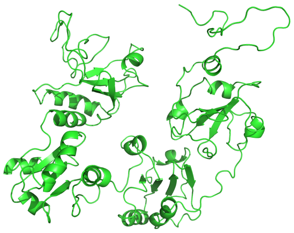	30	0.6
2	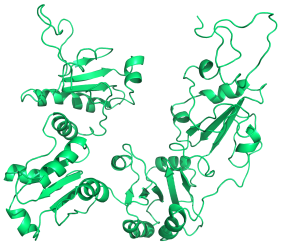	20	0.4
3	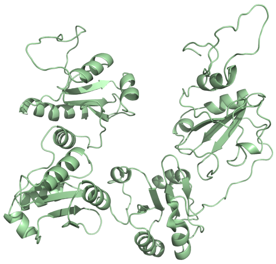	20	0.4
4	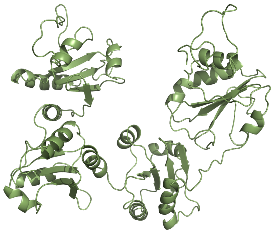	20	0.4
5	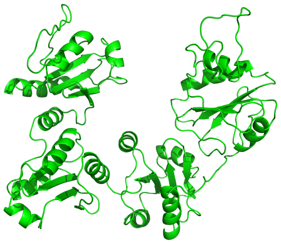	20	0.4
6	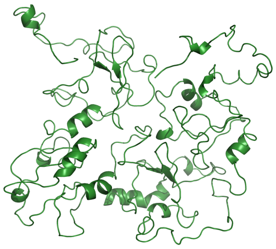	690	13.8
