# Supplementary figures and images for: In Silico Identification of Protein Disulfide Isomerase Gene Families in the De Novo Assembled Transcriptomes of Four Different Species of the Genus Conus
Source: PLoS One. 2016 Feb 9;11(2):e0148390. doi: 10.1371/journal.pone.0148390 (PMC4747531; doi:10.1371/journal.pone.0148390)

**S4 Table. PDI conformations of *C. ximenes* (Cx12).**

|   | Conformation                                                                        | Number of structures | Existence time (ns) |
|---|-------------------------------------------------------------------------------------|----------------------|---------------------|
| 1 | 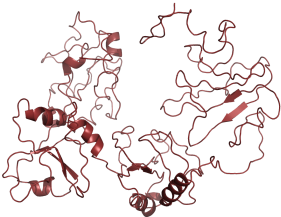   | 30                   | 0.6                 |
| 2 | 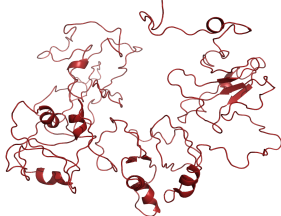   | 20                   | 0.4                 |
| 3 | 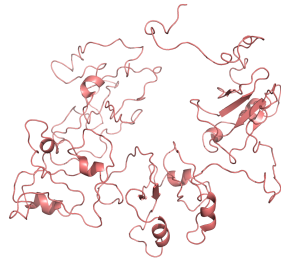  | 20                   | 0.4                 |
| 4 | 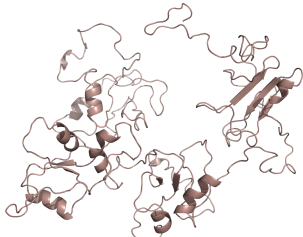 | 20                   | 0.4                 |
| 5 | 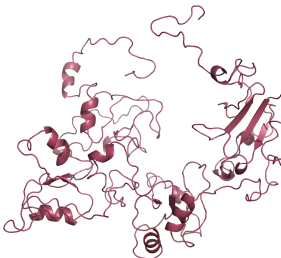 | 20                   | 0.4                 |
| 6 | 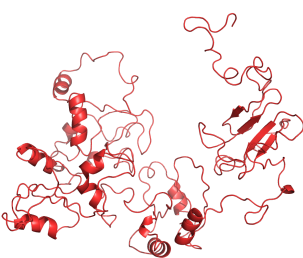 | 690                  | 13.8                |

Supplement: S4 Table — (PDF) [file pone.0148390.s007.pdf]
